# Mapping the knowledge landscape of *Pseudomonas aeruginosa* biofilm-mediated drug resistance: a bibliometric analysis and clinical trial landscape overview

**DOI:** 10.3389/fcimb.2026.1830404

**Published:** 2026-06-01

**Authors:** Peng Zhao, Zejia Wang, Nan Jia, Xin Wang, Jingyu Liu, Yuanqi Zhu

**Affiliations:** 1 Department of Clinical Laboratory, The Affiliated Hospital of Qingdao University, Qingdao, China; 2 Department of Clinical Laboratory, Zibo Infectious Disease Hospital, Zibo, China; 3 School of Basic Medical Sciences, Peking University Health Science Center, Beijing, China; 4 General Office, Zibo Mental Health Center, Zibo, China

**Keywords:** anti-biofilm strategies, antimicrobial resistance, bibliometric analysis, biofilm-mediated drug resistance, clinical trials, *Pseudomonas aeruginosa*

## Abstract

**Background:**

*Pseudomonas aeruginosa* is a major opportunistic pathogen whose ability to form biofilms greatly enhances antimicrobial tolerance and contributes to persistent infection. Although increasing attention has been paid to biofilm-mediated drug resistance, the overall knowledge structure and translational development of this field remain unclear.

**Methods:**

A bibliometric analysis was performed using publications retrieved from the Web of Science Core Collection and Scopus on December 20, 2025. The search covered the period 2014–2025 and focused on *P. aeruginosa*, antimicrobial resistance, and biofilms, resulting in 6,537 publications for bibliometric analysis. To complement the bibliometric findings, a supplementary narrative review of published clinical studies and a separate registered trial landscape overview were conducted. After screening, 6 published clinical studies and 18 registered interventional trials were included.

**Results:**

Global research output on *P. aeruginosa* biofilm-mediated resistance increased steadily from 2014 to 2025. China, the United States, and India were the most productive countries, while the United States showed the leading role in the international collaboration network. Keyword clustering and temporal analyses indicated three major research directions: multidrug resistance evolution and pathogenic synergy, novel antibacterial interventions and functional materials, and clinical translation and efficacy evaluation. The supplementary clinical component showed growing interest in adjunctive and mechanistically targeted strategies, particularly in chronic airway and wound-associated infections, although mature efficacy data remain limited.

**Conclusions:**

Research on *P. aeruginosa* biofilm-mediated drug resistance is shifting from mechanistic exploration toward translational application. This study provides a data-driven overview of the field’s intellectual structure, research hotspots, and emerging trends, and may help guide future anti-biofilm and anti-resistance research.

Systematic review registration:

## 1 Introduction

Antimicrobial resistance (AMR) represents a profound and escalating threat to global health security in the 21st century. According to recent longitudinal projections published in The Lancet, the global annual death toll associated with AMR is forecast to reach 8.22 million by 2050 (Naghavi et al., 2024). Notably, *Pseudomonas aeruginosa* has been designated a “Priority 1: Critical” pathogen by the World Health Organization (WHO), reflecting its high morbidity and formidable repertoire of resistance (Tacconelli et al., 2018; Letizia et al., 2025). Clinically, *P. aeruginosa* is a major cause of healthcare-associated infections, including ventilator-associated pneumonia and catheter-associated urinary tract infections, and these infections may progress rapidly in vulnerable patients (Lieberman and Lieberman, 2003; Ramírez-Estrada et al., 2016; Wang et al., 2019). Furthermore, it remains the central pathogen in chronic pulmonary infections among cystic fibrosis patients (Davies, 2002).

The clinical management of these infections is particularly challenging because *P. aeruginosa* can withstand antimicrobial therapy through a wide range of intrinsic and acquired resistance mechanisms. Intrinsic resistance in *P. aeruginosa* is supported by the low permeability of the outer membrane, constitutive chromosomal determinants such as AmpC β-lactamase, and multidrug efflux systems, whereas acquired resistance arises through target-site mutations, reduced porin permeability, efflux-pump deregulation, and horizontal acquisition of additional resistance genes (Poole, 2011; Castanheira et al., 2021; Qin et al., 2022). However, mounting evidence indicates that biofilm formation further confers enhanced tolerance to antimicrobial agents and host immune defenses (Sharma et al., 2023; Elfadadny et al., 2024), reducing the effectiveness of conventional treatments such as tobramycin and ciprofloxacin (Walters et al., 2003). Besides, although these antibiotics can reduce the number of viable *P. aeruginosa* cells within biofilms, they often fail to eradicate established biofilms (Fernandez-Barat et al., 2017). This incomplete eradication may allow surviving bacteria to persist and regrow, thereby promoting chronic infection and contributing to poor outcomes in patients with cystic fibrosis (Taylor et al., 2014).

Within the biofilm architecture, *P. aeruginosa* embeds itself in a self-produced extracellular matrix composed of exopolysaccharides (Pel, Psl, alginate), extracellular DNA (eDNA), proteins, and lipids, creating a physical and physiological barrier that restricts antibiotic penetration and fosters metabolic heterogeneity (Ghafoor et al., 2011; Thi et al., 2020). Cells within biofilms may require antibiotic concentrations 10–1000 times higher than planktonic cells, which are free-living bacterial cells and not associated with biofilms, to achieve inhibitory or bactericidal effects (Mah and O’Toole, 2001). Compared with their planktonic counterparts, biofilm-embedded populations are exposed to oxygen and nutrient gradients, local hypoxia, redox stress, and slowed growth (Ciofu and Tolker-Nielsen, 2019). These microenvironmental conditions enrich persister cells, promote stress-response programs, and increase apparent minimum inhibitory concentration (MIC) values in biofilm-based susceptibility testing as well as minimum biofilm eradication concentrations (MBECs) relative to genetically comparable planktonic cells (Elmanama et al., 2020). Furthermore, the matrix can sequester antibiotics and induces resistance to treatment (Suci et al., 1994). The regulation of biofilm development is intricately linked to quorum sensing (QS) systems (Ambreetha and Singh, 2023), two-component regulatory systems (Valentini and Filloux, 2016), and the second messenger cyclic di-GMP (c-di-GMP) (Kuchma et al., 2015), all of which coordinate virulence expression and structural maturation. Consequently, the biofilm-mediated resistance necessitates re-evaluation of dosing strategies and treatment paradigms.

In response to these challenges, therapeutic innovation has shifted toward anti-virulence and anti-biofilm strategies that aim to disarm the pathogen rather than kill it. Promising approaches include QS inhibitors (Flores-Maldonado et al., 2024), c-di-GMP modulators (Liu et al., 2018; Cordery et al., 2024), bacteriophage therapy (Wang et al., 2024), antimicrobial peptides (Furusawa et al., 2016), nanoparticles (Wusiman et al., 2022; Palau et al., 2023), monoclonal antibodies (Patel et al., 2017) and vaccines (Yang et al., 2017). These strategies seek to disrupt biofilm integrity, enhance antibiotic penetration, and restore host immune clearance, thereby overcoming the limitations of conventional antimicrobials (Ramesh et al., 2025). Despite these advances, the high antigenic variability and genetic plasticity of *P. aeruginosa* continue to impede broad-spectrum efficacy.

Given the accelerating volume of research on *P. aeruginosa* biofilm-mediated resistance, there is a growing need to systematically map the intellectual structure, thematic evolution, and collaborative dynamics of this scientific domain. Bibliometric analysis offers a powerful, quantitative approach to visualize the knowledge landscape by analyzing publication trends, citation networks, keyword co-occurrence, and institutional contributions. Such studies enable identification of research hotspots, emerging fronts, and gaps in the literature, thereby guiding future investigations and funding priorities, which have already been extensively applied to other microorganisms (Shi, 2025; Tian et al., 2025). While numerous reviews have synthesized the biological and clinical aspects of *P. aeruginosa* biofilms (Hanot et al., 2025; Wang et al., 2025; Elbehiry et al., 2026), a comprehensive bibliometric assessment of this field remains lacking. This study aims to fill this gap by conducting a visualized, data-driven exploration of the global research output on *P. aeruginosa* biofilm-mediated drug resistance, providing a strategic overview of the field’s development and trajectory.

## 2 Materials and methods

### 2.1 Search strategy for bibliometric analysis

**Figure 1:**
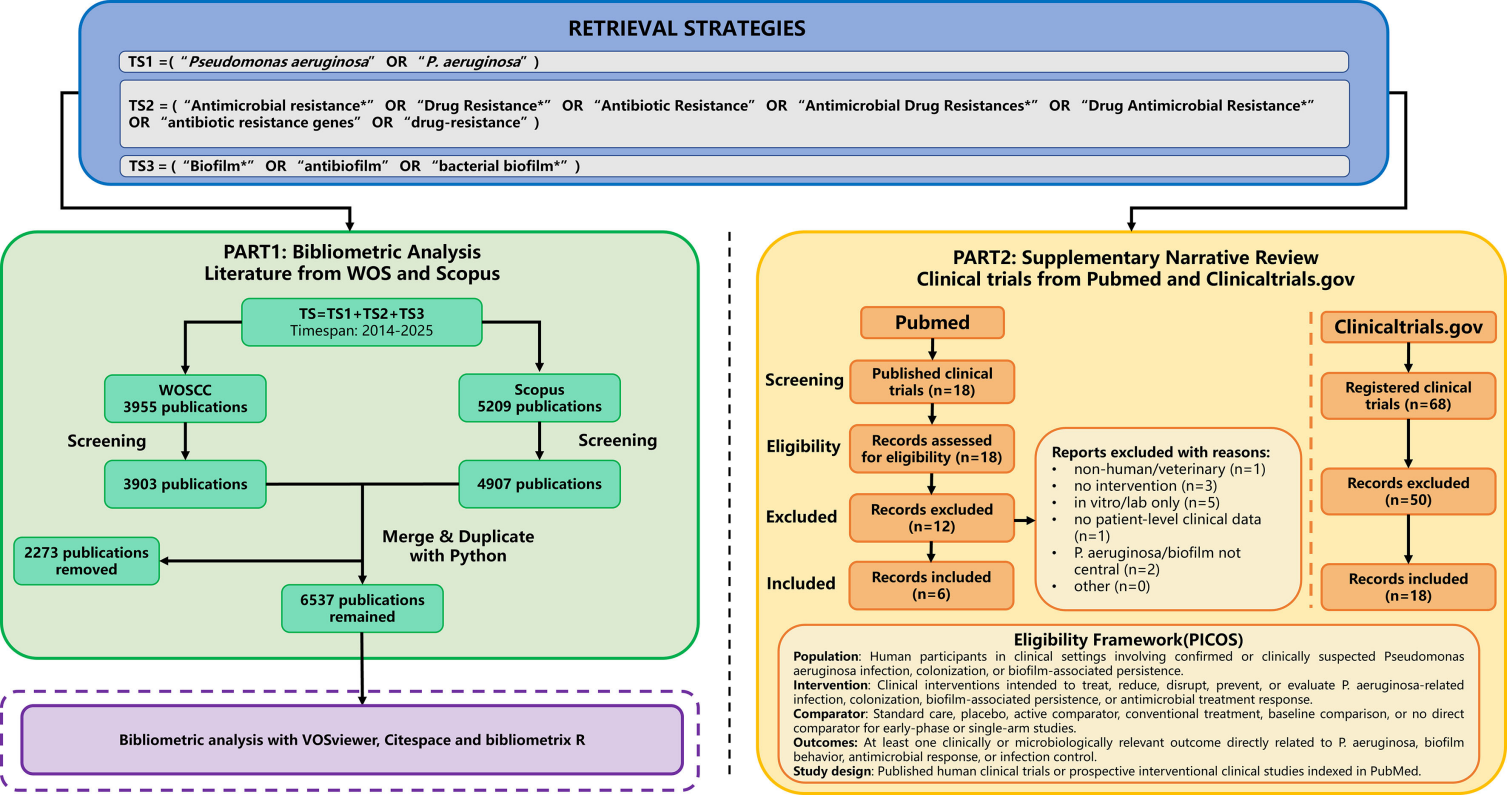
Workflow of literature retrieval, screening, and analysis. The study was conducted in two parts. Part 1 comprised a bibliometric analysis of publications retrieved from Web of Science Core Collection (WOSCC) and Scopus using the combined search strategy TS = TS1 + TS2 + TS3 over the period 2014–2025. After screening, 3,903 records from WOSCC and 4,907 records from Scopus were retained, and duplicate removal using Python yielded 6,537 publications for bibliometric analysis with VOSviewer, CiteSpace, and the bibliometrix R package. Part 2 consisted of a narrative review of clinical trials identified from PubMed and ClinicalTrials.gov. 6 published and 18 registered records were finally included after screening and eligibility assessment.

A comprehensive literature search was conducted on December 20, 2025, using the Web of Science Core Collection (WoSCC) and Scopus (**Figure 1**). The use of these two databases was intended to improve data coverage and reduce database-specific bias in the bibliometric analysis. The search strategy was designed to capture publications related to *P. aeruginosa*, antimicrobial resistance, and biofilms. The core search terms can be found in **Supplementary Material** (**Supplementary Table 1**), with the publication timespan restricted to 2014–2025. The language is restricted to English, which may have introduced potential language bias by excluding relevant studies published in other languages. However, because English is the predominant language of international biomedical publication and because WoSCC and Scopus primarily index English-language records, this restriction was considered unlikely to substantially affect the overall bibliometric patterns.

The initial retrieval yielded 3,955 records from WoSCC and 5,209 records from Scopus. For bibliometric purposes, both original research articles and review articles were retained because original studies reflect primary knowledge production, whereas review articles capture evidence synthesis, agenda setting, and intellectual consolidation within the field. Therefore, including both article types can enrich citation and thematic analyses. After this initial screening, 3,903 WoSCC records and 4,907 Scopus records were retained. After deduplication with Python, a total of 6,537 publications remained for bibliometric analysis. Bibliographic information and cited-reference data were then imported into VOSviewer (version 1.6.18) (van Eck and Waltman, 2010), CiteSpace (version 6.2.R2) (Synnestvedt et al., 2005), and the bibliometrix R package (version 5.0) (Aria and Cuccurullo, 2017) for further analysis and visualization.

### 2.2 Supplementary clinical trials review

To complement the bibliometric analysis with clinically relevant translational evidence, we conducted a supplementary narrative review of published clinical studies from PubMed and a separate registered trial landscape overview using ClinicalTrials.gov. The PubMed component was designed to summarize peer-reviewed clinical evidence, whereas ClinicalTrials.gov records were used only to characterize the translational development pipeline and were not treated as peer-reviewed efficacy evidence. In contrast to the bibliometric search, this component focused on the intersection between *P. aeruginosa* and biofilm-related clinical research.

For the PubMed search, the strategy was adapted to PubMed syntax, with the publication period limited to 2014–2025 and records restricted to clinical trials. This design was adopted to increase sensitivity for clinically oriented biofilm studies, because many interventional studies do not explicitly include antimicrobial-resistance terminology in their titles or abstracts. The PubMed search identified 18 clinical trial records. To characterize the registered translational development pipeline, ClinicalTrials.gov was searched over the same time period (2014–2025) using the condition term “*Pseudomonas aeruginosa*,” with the study type restricted to interventional studies. This search yielded 68 registered clinical trials.

The titles, abstracts, and registry information of records retrieved from both sources were manually screened according to predefined PICOS-informed eligibility criteria (**Supplementary Table 2**). Studies were included if they were human clinical trials or prospective interventional studies involving *P. aeruginosa* infection, colonization, or clinically relevant detection in a biofilm-related setting, and if they evaluated an intervention affecting clinical outcomes, microbial burden, biofilm composition, or biofilm-associated therapeutic response.

Studies were excluded if they were: (1) non-human or veterinary studies; (2) environmental or engineering studies without patient-level clinical data; (3) observational or descriptive studies without intervention; (4) purely *in vitro* or laboratory-based analyses; or (5) studies in which *P. aeruginosa* or biofilm was not a central clinical component. After screening, 12 PubMed records and 50 ClinicalTrials.gov records were excluded. The remaining PubMed-indexed published clinical studies were included in the supplementary narrative review and subsequently underwent methodological quality and risk-of-bias assessment using study-design-appropriate tools. Individually randomized parallel-group trials were evaluated using the revised Cochrane risk-of-bias tool for randomized trials (RoB 2) (Sterne et al., 2019), the crossover study was evaluated using the RoB 2 crossover-trial version, and the single-arm prospective phase I/II study was assessed using the NIH Quality Assessment Tool for Before-After Studies With No Control Group. Registered interventional trials from ClinicalTrials.gov were summarized separately as a trial landscape overview and were not subjected to formal risk-of-bias assessment because registry records do not constitute peer-reviewed clinical outcome evidence.

## 3 Results

### 3.1 Analysis of publication trends

**Figure 2:**
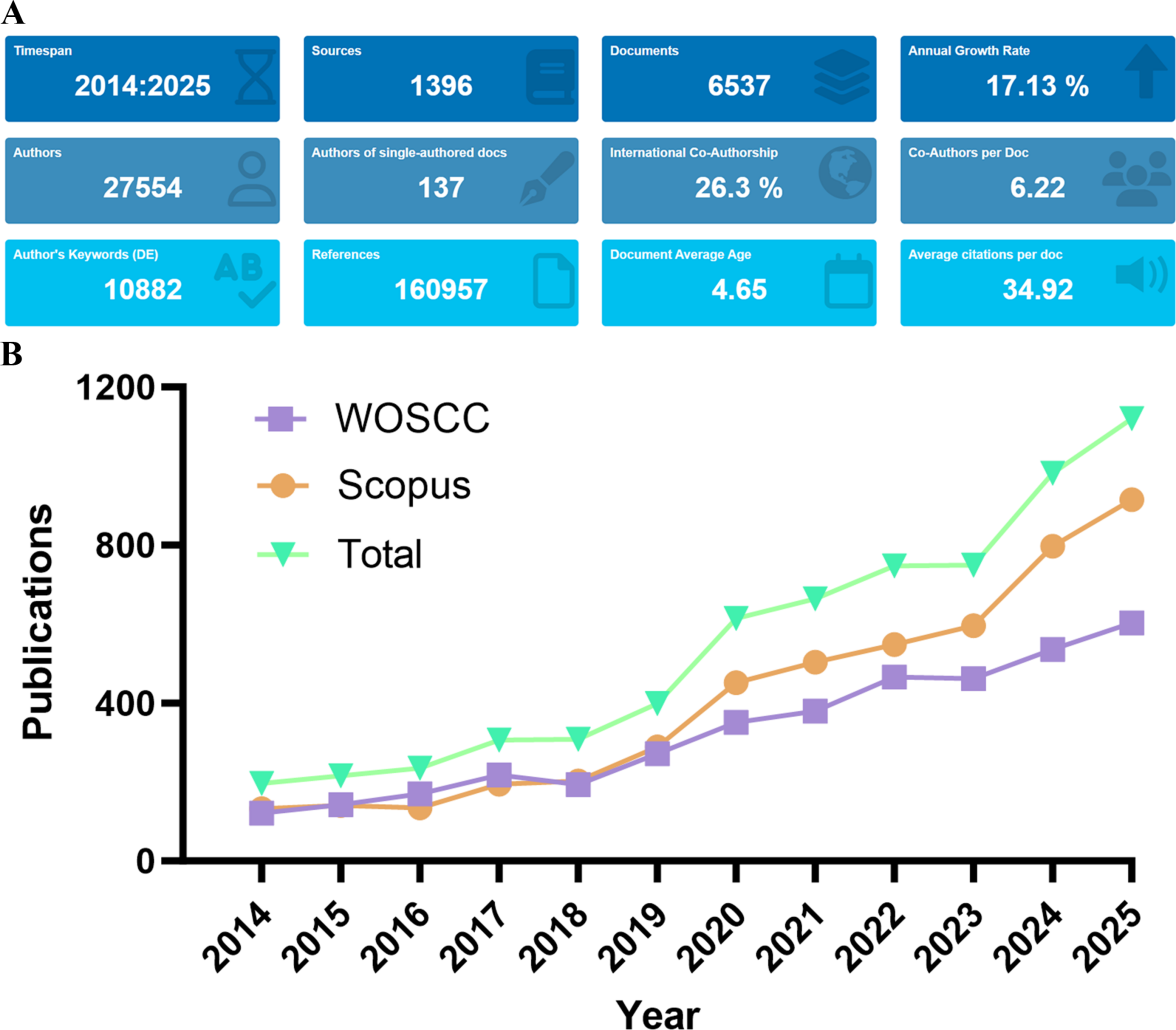
Bibliometric analysis and publication trends of *P. aeruginosa* biofilm-mediated resistance (2014–2025). **(A)** core bibliometric indicators of the merged WoSCC–Scopus dataset. **(B)** Annual and cumulative publication growth from 2014 to 2025.

Using the merged WoSCC–Scopus dataset (n = 6,537), research output in this field showed a sustained upward trajectory from 2014 to 2025 (**Figure 2**). The cumulative publication curve indicates a clear acceleration over time, highlighting the rapid expansion of this research area.

### 3.2 Analysis of countries

**Figure 3:**
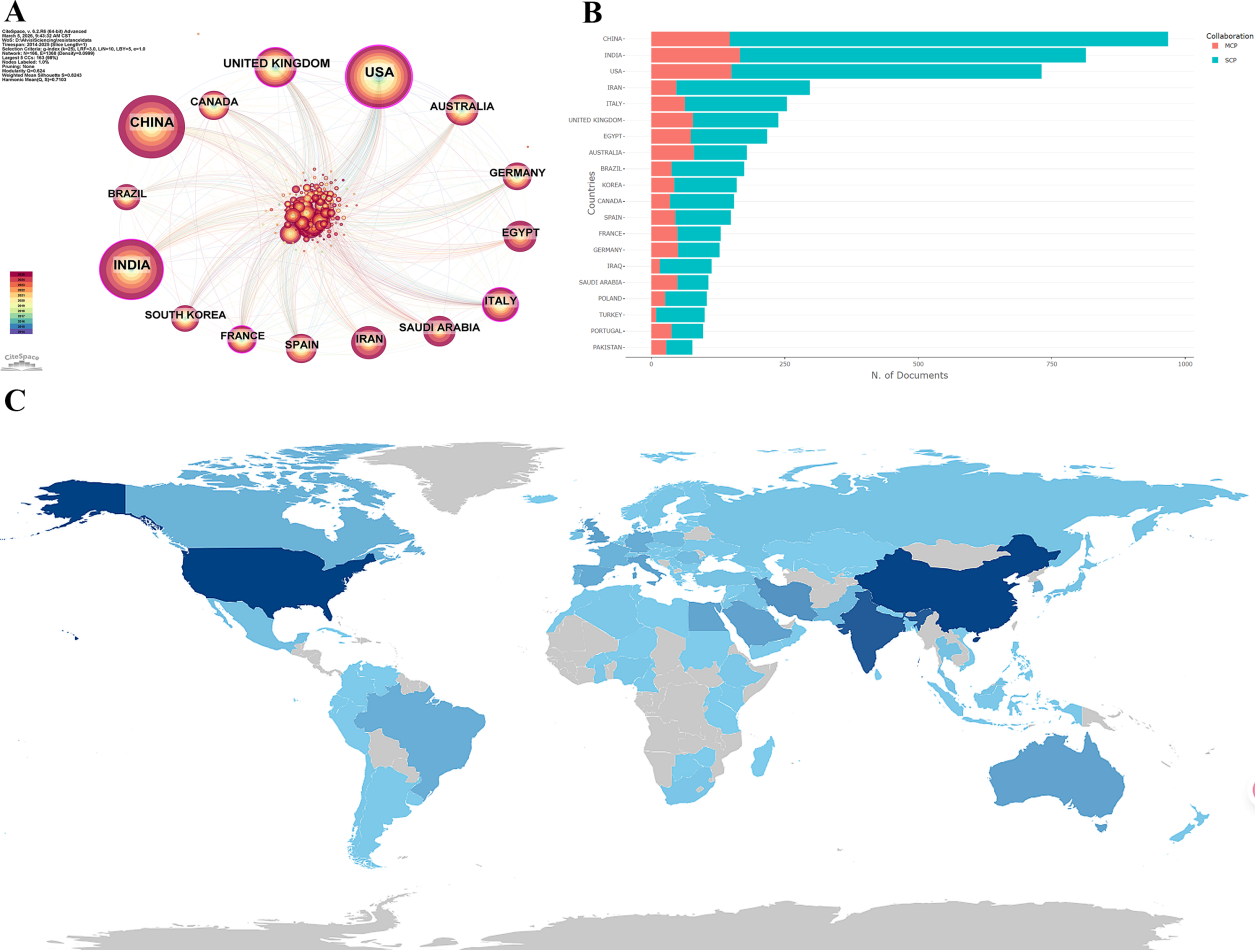
Global distribution and collaboration networks of research. **(A)** country co-occurrence network: node size reflects publication frequency. The purple outer rings denote countries with centrality > 0.1, identifying them as core intellectual bridges in the global network. **(B)** research output by corresponding author’s country: comparison of publication volumes, distinguished by single-country (SCP) and multi-country (MCP) publications based on the affiliation of the corresponding author. **(C)** geographic distribution: world map illustrating the global density of research output on (P) aeruginosa biofilm resistance.

The global research landscape is dominated by China (n = 1,081), the USA (n = 1,030), and India (n = 937), which contributed the highest publication volumes (**Figure 3**; **Table 1**). Betweenness centrality—a network metric indicating the extent to which a unit acts as a bridge connecting different parts of the collaboration network—was highest for the United States (0.47), followed by India (0.14); the United Kingdom and Italy also showed notable bridging roles (0.12 each). While the USA exhibit a greater proportion of multi-country publications (MCP), research output from China is characterized by a higher frequency of single-country publications (SCP), reflecting a more domestically concentrated research focus despite their high productivity.

**Table 1:** Top 10 most productive countries.

Country	Freq	Centrality	Year
China	1081	0.09	2014
USA	1030	0.47	2014
India	937	0.14	2014
UK	394	0.12	2014
Italy	325	0.12	2014
Iran	322	0.03	2014
Egypt	282	0.03	2014
Saudi Arabia	270	0.08	2014
Australia	255	0.04	2014
Spain	245	0.09	2014

### 3.3 Analysis of affiliates

**Figure 4:**
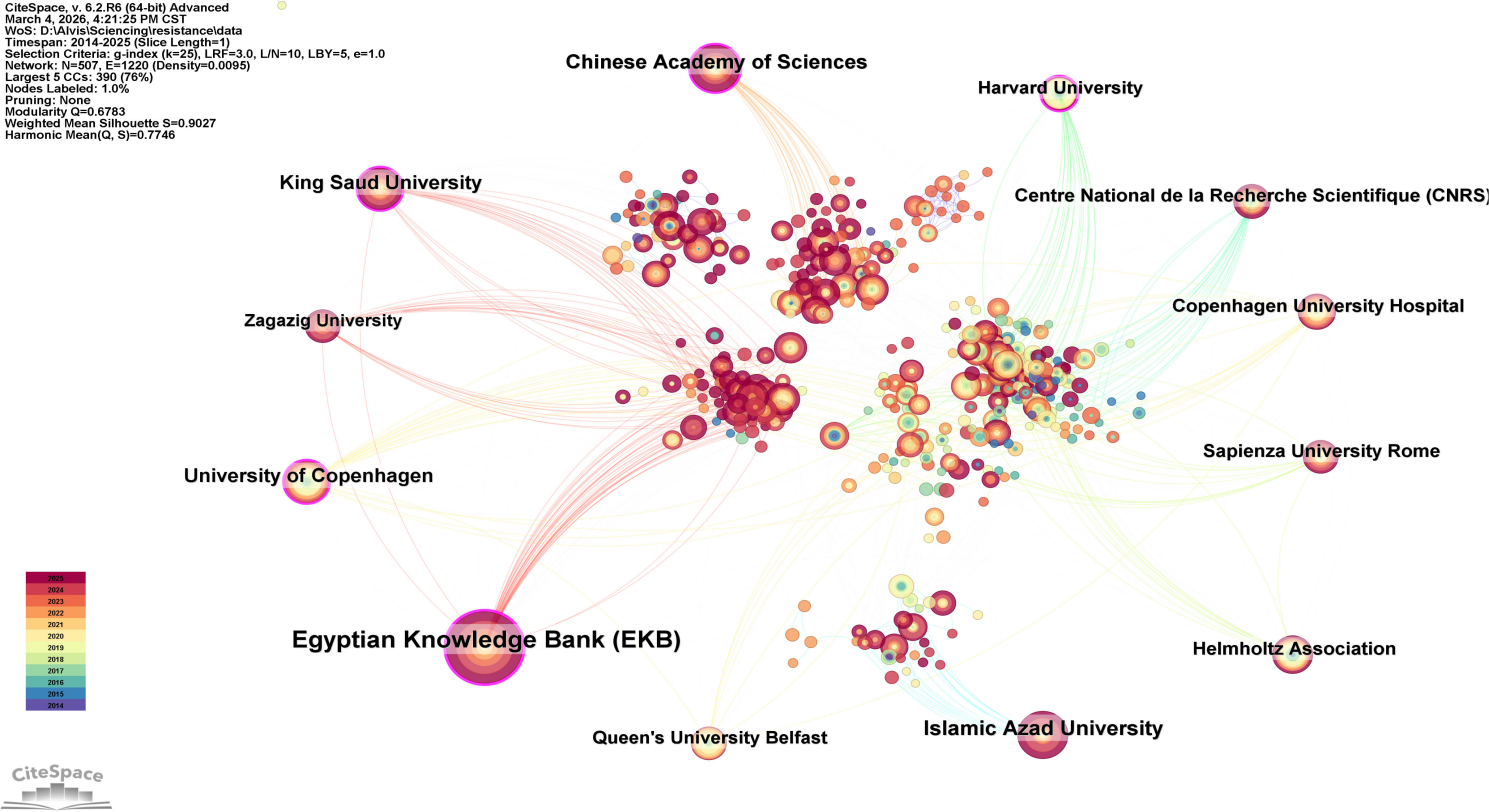
Institutional collaboration network. Visualization of inter-institutional partnerships, highlighting the Egyptian knowledge bank (n = 167) as the most prolific institution. Other visual encodings are consistent with those in **Figure 3A**.

The Egyptian Knowledge Bank occupies the central position in the research network, exhibiting both the highest publication frequency (n=167) and the greatest betweenness centrality (0.22) (**Figure 4**; **Table 2**). Highly productive organizations such as the Islamic Azad University (n=80) and Chinese Academy of Sciences (n=74) also emerge as critical research hubs with significant structural influence. The institutional co-occurrence map illustrates a well-connected global ecosystem where established entities like Harvard University and Copenhagen University Hospital collaborate with emerging research groups to drive the study of *P. aeruginosa* biofilm resistance.

**Table 2:** Top 10 most productive affiliates.

Affiliate		Freq	Centrality	Year
Egyptian Knowledge Bank (EKB)	167		0.22	2015
Islamic Azad University	80		0.04	2017
Chinese Academy of Sciences	74		0.12	2016
University of Copenhagen	63		0.11	2014
King Saud University	60		0.14	2016
Copenhagen University Hospital	50		0.03	2014
Helmholtz Association	49		0.06	2014
Centre National de la Recherche Scientifique (CNRS)	47		0.07	2014
Sapienza University Rome	46		0.05	2014
Harvard University	41		0.14	2014

### 3.4 Analysis of authors

**Figure 5:**
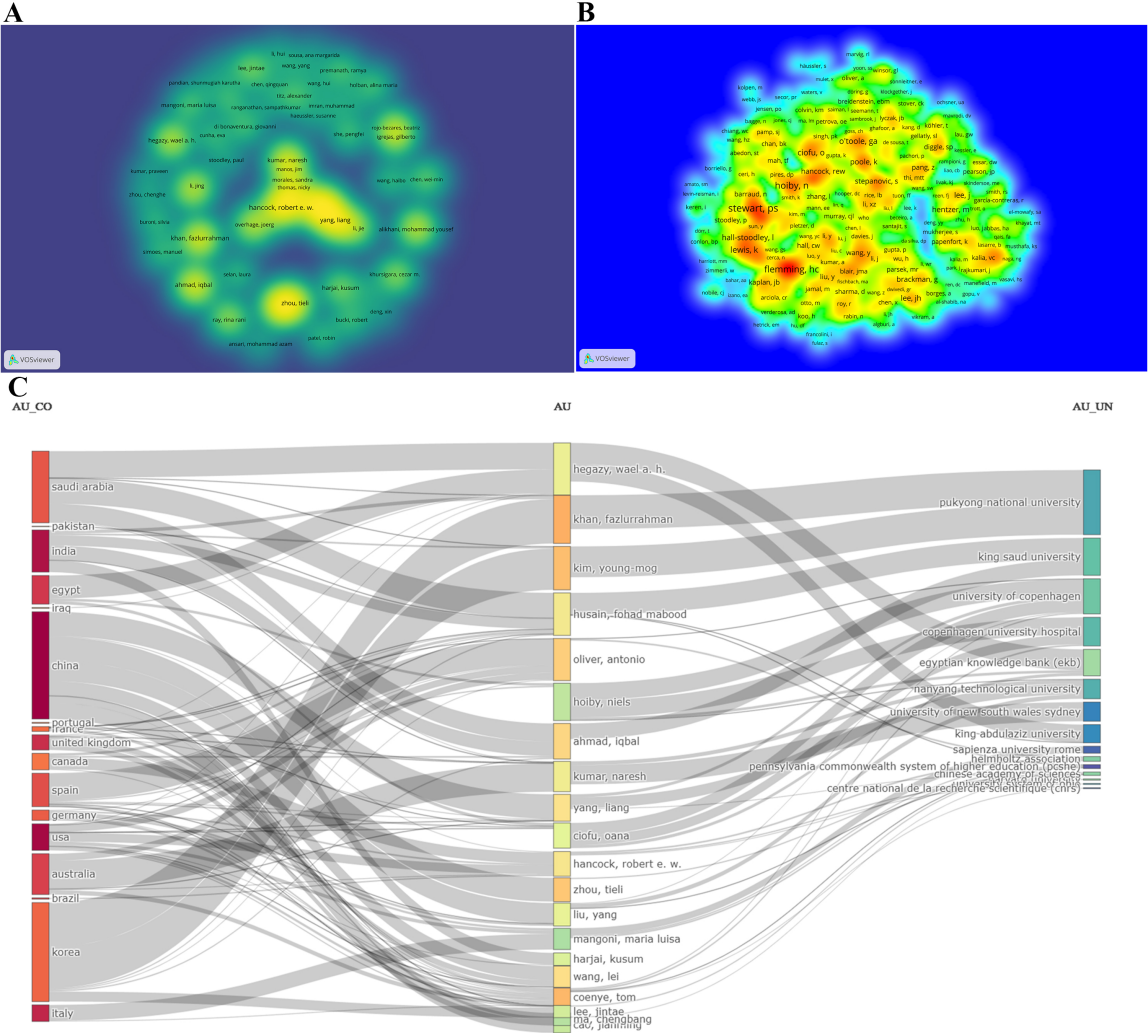
Author productivity and collaboration patterns. **(A)** Density map of author productivity **(B)** Author co-citation density map **(C)** Three-field plot: Illustrates the thematic and collaborative links between countries (left), authors (middle), and affiliations (right).

The author analysis reveals a prolific and collaborative community, with Hancock, Robert E. W. (29 documents) and Khan, Fazlurrahman (27 documents) leading in total output (**Figure 5**; **Table 3**). Academic influence is most concentrated in the works of Hancock, Robert E. W. (2,955 citations) and Ciofu, Oana (2,260 citations), who serve as the field’s primary intellectual pillars. The co-authorship network underscores a decentralized yet well-connected structure, where these key figures act as central hubs for diverse research clusters spanning mechanistic, clinical, and biotechnological domains.

**Table 3:** Top 15 most productive authors.

Author	Documents	Citations
Hancock, Robert E. W.	29	2955
Khan, Fazlurrahman	27	813
Coenye, Tom	25	1920
Kim, Young-Mog	23	741
Zhou, Tieli	23	381
Oliver, Antonio	21	1302
De La Fuente-Núñez, César	20	2046
Wang, Lei	20	565
Yang, Liang	20	534
Ahmad, Iqbal	20	481
Kumar, Naresh	19	749
Husain, Fohad Mabood	19	424
Hegazy, Wael A. H.	18	526
Liu, Yang	18	427
Ciofu, Oana	17	2260

### 3.5 Analysis of journals

**Figure 6:**
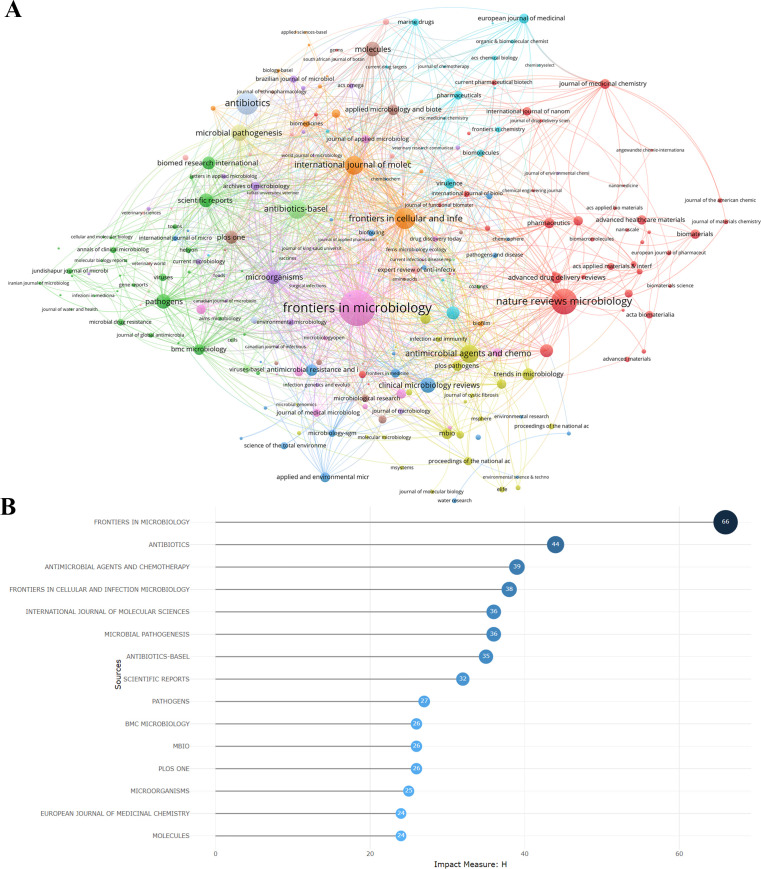
Journal impact and co-citation analysis. **(A)** journal co-citation network: nodes represent journals **(B)** top 10 journals with the highest h-index: comparison of journals by local h-index, led by Frontiers in Microbiology.

Research in this field is distributed across 1396 distinct sources, with Frontiers in Microbiology identified as the most prolific journal, contributing 379 documents and 18,250 total citations (**Figure 6**; **Table 4**). It is also the journal with the highest h-index (66). The impact factors (IF) of these leading journals range from 3.3 to 4.9, indicating that research in this field is mainly disseminated through journals with moderate to high academic influence.

**Table 4:** Top 10 journals with the highest H-index.

Journal	H-index	Documents	Citations	IF
Frontiers in Microbiology	66	379	18250	4.5
Antibiotics	44	208	7194	4.6
Antimicrobial Agents and Chemotherapy	39	103	4882	4.5
Frontiers in Cellular and Infection Microbiology	38	134	6283	4.8
International Journal of Molecular Sciences	36	120	5081	4.9
Microbial Pathogenesis	36	163	3856	3.5
Antibiotics-Basel	35	137	4909	4.6
Scientific Reports	32	85	2681	3.9
Pathogens	27	78	3086	3.3
BMC Microbiology	26	92	1701	4.2

### 3.6 Analysis of keywords

**Figure 7:**
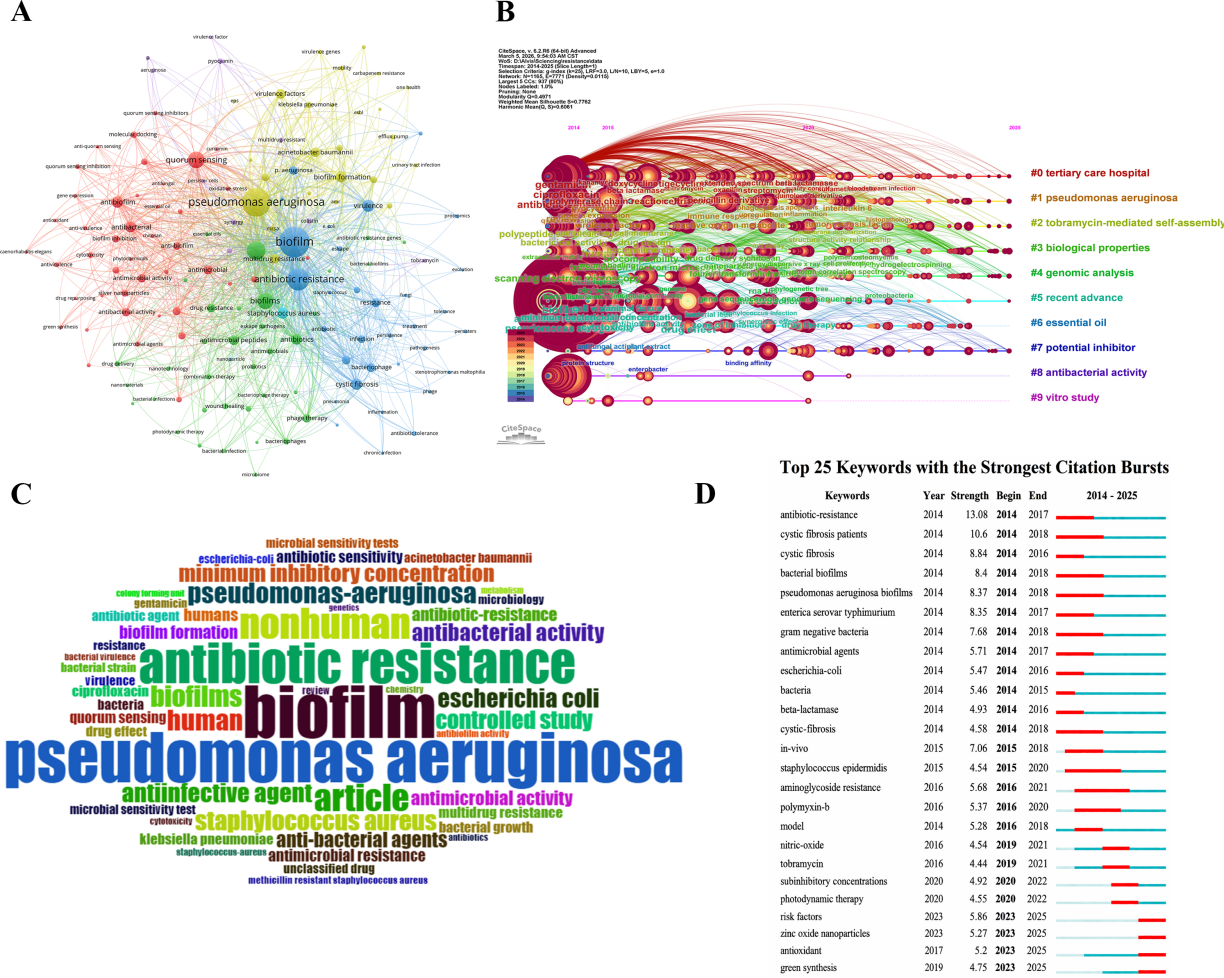
Keyword co-occurrence, clustering, and thematic evolution of (P) aeruginosa biofilm-resistance research. **(A)** keyword co-occurrence network: visualization of high-frequency terms; node size reflects frequency and colors represent distinct thematic clusters. **(B)** timeline view of keyword clusters: illustrates the temporal progression of 10 major thematic clusters (labeled #0–#9) from 2014 to 2025, tracking the field’s shift toward clinical translation and nanotechnology. **(C)** keyword word cloud: graphical representation of the most prominent research terms. **(D)** top 20 keywords with the strongest citation bursts: Red segments indicate periods of peak research interest. The “cystic fibrosis” burst (18.54) marks the foundational period, while “green synthesis” and “ *Chromobacterium violaceum*” signify current frontiers (2023–2025).

Based on the keyword co-occurrence network, timeline clustering, word cloud, and citation burst analysis (**Figure 7**), together with the thematic classification presented in **Table 5**, *P. aeruginosa* biofilm resistance research can be organized into three major research directions: the evolution of multidrug resistance (MDR) and pathogenic synergy (Clusters #1 and #4), novel antibacterial interventions and functional materials (Clusters #2, #3, #5, #6, and #7), clinical translation and efficacy evaluation (Clusters #0, #8, and #9).

**Table 5:** Core research dimensions and thematic clustering of P. aeruginosa biofilm resistance studies.

Rank	Research direction	Cluster ID	Representative keywords
1	Multidrug Resistance (MDR) Evolution and Pathogenic Synergy	#1, #4	Pseudomonas aeruginosa, biofilm, synergistic effect, drug therapy, whole genome sequencing, phylogeny / 16S rRNA
2	Novel Antibacterial Interventions and Functional Materials	#2, #3, #5, #6, #7	tobramycin-mediated self-assembly, essential oil, plant extract, potential inhibitor, hydrogel, electrospinning, chitosan, biocompatibility / wound healing
3	Clinical Translation and Efficacy Evaluation	#0, #8, #9	tertiary care hospital, bloodstream infection, β-lactamase / ESBL, ciprofloxacin / gentamicin, antibacterial activity, *in vitro* study

Citation burst strength refers to the magnitude of an abrupt increase in attention to a keyword over a defined period and therefore reflects the intensity of emerging scholarly interest. From a temporal perspective, the field shows a clear stage-based evolution. During 2014–2018, the strong citation burst of “cystic fibrosis” (strength = 18.54) marked the foundational phase, underscoring the clinical relevance of biofilm-associated chronic infection. In the intermediate stage, roughly 2016–2022, research attention moved toward mechanistic and therapeutic challenges, with burst terms such as antibiotic tolerance, quorum quenching, nitric oxide, and antimicrobial susceptibility, reflecting intensified interest in resistance regulation, quorum-sensing interference, and anti-biofilm intervention targets. More recently, in 2023–2025, “green synthesis” and “Chromobacterium violaceum” emerged as frontier keywords, suggesting that current research is increasingly oriented toward sustainable synthesis strategies, natural-source inhibitory approaches, and innovative material-based therapies against biofilm resistance.

### 3.7 Clinical trials analysis

After screening of 18 PubMed clinical-trial records, 6 published studies were included as directly relevant to *Pseudomonas aeruginosa* biofilm-related clinical research (**Supplementary Table 3**). These studies covered three main settings: chronic wounds, catheter-related bloodstream infection, and cystic fibrosis airway infection. Most adopted randomized or crossover designs, with one prospective phase I/II interventional study. Methodological quality and risk-of-bias assessment showed that two randomized trials were judged to have low risk of bias, three randomized or crossover studies raised some concerns, and the single-arm prospective phase I/II study was rated as fair methodological quality. These assessments indicate that the published clinical evidence remains exploratory and should be interpreted cautiously.

In addition to the published studies, 18 registered interventional trials relevant to *P. aeruginosa* biofilm-associated infection were identified from ClinicalTrials.gov (**Supplementary Table 4**). These registered studies were concentrated mainly in chronic pulmonary infection, particularly in cystic fibrosis and non-cystic fibrosis bronchiectasis, with additional trials involving surgical-site infection, pressure ulcers, diabetic foot ulcers, burn wounds, and prevention of nosocomial pneumonia. The intervention landscape was dominated by nontraditional anti-Pseudomonas strategies, especially bacteriophage-based therapies, alongside biofilm- or virulence-targeting monoclonal antibodies, inhaled nitric oxide formulations, and gallium-based approaches. Of the 18 registered studies, 10 were listed as completed or study complete, 3 were recruiting, 1 was discontinued by the sponsor, and 4 had unclear, overdue, or stale registry status. Overall, the registered-trial landscape indicates a strong translational emphasis on adjunctive approaches aimed at reducing bacterial persistence, disrupting biofilm-associated infection, or preventing recurrent pulmonary exacerbations, although mature efficacy data remain unavailable for many of these interventions.

Together, the published clinical evidence and registry-based trial landscape suggest that current translational research on *P. aeruginosa* biofilm-related infection is shifting from conventional antimicrobial comparison alone toward adjunctive and mechanistically targeted strategies, particularly in chronic airway and wound-associated settings.

## 4 Discussion

### 4.1 Overall analysis

This study combined bibliometric analysis with a supplementary review of both published clinical trials and registered interventional studies to characterize the knowledge structure and translational trajectory of *Pseudomonas aeruginosa* biofilm-mediated drug resistance research. From 2014 to 2025, the field expanded steadily and developed around three interconnected themes: multidrug resistance (MDR) evolution and pathogenic synergy, novel antibacterial interventions and functional materials, and clinical translation and efficacy evaluation. Country-, institution-, and author-level analyses indicate that this is now a mature and multidisciplinary field, with the United States occupying the strongest bridging position, while China and India contribute the largest publication outputs. At the intellectual level, authors such as Hancock Robert E. W.

Khan Fazlurrahman and Ciofu Oana represent influential yet complementary research programs spanning antibiofilm materials, antimicrobial peptides, and resistance adaptation under biofilm conditions.

An important strength of the present bibliometric structure is that it maps well onto identifiable lines of representative literature rather than abstract keyword clusters alone. A major cluster led by Fazlurrahman Khan at Pukyong National University focuses on the application of nanoparticles (Au, Ag, ZnO) for biofilm inhibition (Khan et al., 2021; Tabassum et al., 2024a, 2024b; Fattahi et al., 2025). Another prominent cluster centered around Robert E. W. Hancock emphasizes the engineering of synthetic antibiofilm peptides and polymers (Fuente-Núñez et al., 2014; Bionda et al., 2016; Ma et al., 2018; Etayash et al., 2020), alongside the standardization of assessment methodologies (Haney et al., 2021). Meanwhile, the cluster formed by Oana Ciofu and Niels Hoiby investigates the evolutionary adaptation of *P. aeruginosa* under antibiotic stress, highlighting the critical role of biofilms in driving AMR (Fernandez-Barat et al., 2017; Ahmed et al., 2018; Higazy et al., 2024a). Together, these examples indicate that the field is organized not only by topic, but also by relatively stable translational agendas.

### 4.2 Research hotspots of *P. aeruginosa* biofilm-mediated resistance

In the present study, keyword co-occurrence, timeline clustering, word-cloud analysis, and keyword burst detection converged on three major directions: multidrug resistance (MDR) evolution and pathogenic synergy, novel antibacterial interventions and functional materials, and clinical translation and efficacy evaluation. This structure indicates that research on *P. aeruginosa* biofilm-mediated resistance has progressed from defining core resistance mechanisms toward developing anti-biofilm strategies and testing their clinical applicability.

In particular, Clusters #1 and #4, represented by terms such as “*Pseudomonas aeruginosa*,” “biofilm,” “synergistic effect,” “drug therapy,” “whole-genome sequencing,” and “phylogeny/16S rRNA,” reflect the mechanistic foundation of the field. Clusters #2, #3, #5, #6, and #7, including “tobramycin-mediated self-assembly,” “essential oil,” “plant extract,” “potential inhibitor,” “hydrogel,” “electrospinning,” “chitosan,” and “biocompatibility/wound healing,” indicate a growing intervention-oriented branch. Clusters #0, #8, and #9, characterized by “tertiary care hospital,” “bloodstream infection,” “β-lactamase/extended-spectrum β-lactamase (ESBL),” “ciprofloxacin/gentamicin,” “antibacterial activity,” and “*in vitro* study,” suggest increasing attention to clinical evaluation and translational validation. The following subsections therefore discuss these three result-derived dimensions while integrating mechanistic and clinical evidence from representative studies.

#### 4.2.1 MDR evolution and pathogenic synergy

The prominence of MDR- and biofilm-related keywords suggests that resistance evolution remains the mechanistic core of this field. The evolution of MDR in *P. aeruginosa* biofilms is a synergistic process driven by chromosomal mutations, horizontal gene transfer (HGT), and microenvironmental stress. Biofilm-specific pathways, including RpoS-mediated stationary phase and Anr-regulated hypoxic stress, can facilitate rapid resistance to ciprofloxacin (Stewart et al., 2015). Under sub-inhibitory antibiotic pressure, the biofilm matrix promotes low-level mutational resistance (Ahmed et al., 2018), with *in vivo* ciprofloxacin resistance emerging as early as the second generation via mutations in *nfxB*, *mexZ*, and *parS* (Higazy et al., 2024b). These observations help explain why terms related to antibiotic tolerance, persister cells, and antimicrobial susceptibility appeared as important temporal signals: biofilm-mediated resistance is not only a stable genetic phenotype but also a dynamic survival state shaped by local stress and treatment exposure.

HGT provides another mechanistic explanation for the clustering of genomic and resistance-related terms. Biofilm-derived outer membrane vesicles can serve as DNA-shielded vehicles for plasmid transfer, thereby facilitating the dissemination of resistance determinants within dense bacterial communities (Johnston et al., 2023). IncP-2 type plasmids are also relevant to MDR dissemination because they can carry combinations of resistance genes that broaden the resistance phenotype (Mei et al., 2024). Phage-mediated HGT is equally noteworthy, with recent work showing that phages participating in the horizontal transfer of efflux pump regulatory genes, such as mexT, mexR, and nfxB (Wu et al., 2025).

The keyword pattern also highlights pathogenic synergy within polymicrobial biofilms. Interspecies interactions can modulate antimicrobial sensitivity through metabolite exchange, QS signaling and changes in biofilm architecture, typically reducing therapeutic efficacy (Orazi and O’Toole, 2019). *P. aeruginosa* can sense diffusible signal factors via the PA1396 histidine kinase, leading to altered biofilm structure and increased tolerance (An et al., 2019). *Candida tropicalis* supernatants have also been reported to upregulate *P. aeruginosa* genes such as *aph(3’)-IIb* and *gyrA*, enhancing resistance to aminoglycosides and fluoroquinolones (Sachdeva et al., 2025). In cystic fibrosis, which emerged as a strong early burst keyword, the biofilm matrix blocks antibiotic diffusion, while efflux systems including MexAB-OprM and MexCD-OprJ contribute to biofilm formation and azithromycin resistance (Sherrard et al., 2014). Interspecies competition for nutrients induces ATP depletion, further enhancing antibiotic tolerance (Nabb et al., 2019).

These community-level effects are particularly important because *P. aeruginosa* frequently coexists with other microorganisms in chronic wounds, airways, and device-associated infections. In *P. aeruginosa*–*S. aureus* biofilms, AmpC β-lactamase, exopolysaccharides (Psl and Pel), and quorum-sensing regulators (LasR and RhlR) shield S. aureus from β-lactam antibiotics (da Cruz Nizer et al., 2026). Thus, the bibliometric prominence of biofilm, synergistic effect, cystic fibrosis, and genomic-analysis-related terms reflects a broader conceptual shift: *P. aeruginosa* biofilm-mediated resistance is increasingly understood as an adaptive, community-level, and ecologically regulated process rather than a single-species or single-gene resistance phenotype. This interpretation also explains why treatment strategies designed for monomicrobial planktonic infection may be insufficient for biofilm-associated polymicrobial disease (Al-Wrafy et al., 2023).

#### 4.2.2 Novel antibacterial interventions and functional materials

The second major direction identified by keyword clustering was the development of novel antibacterial interventions and functional materials. Effective interventions often need to disrupt matrix barriers, improve local drug penetration, interfere with bacterial communication, or combine antimicrobial activity with tissue repair.

Chitosan-based antimicrobial hydrogels and electrospun fibrous dressings represent one clinically relevant extension of this material-oriented branch, particularly for *P. aeruginosa* biofilm-associated wound infection. The protonated amino groups of chitosan can interact electrostatically with negatively charged bacterial membranes, thereby inducing membrane damage and intracellular leakage; the MIC against *P. aeruginosa* is typically 0.5–2 mg/mL (Matica et al., 2019). By means of chemical crosslinking or physical blending, chitosan hydrogels can be endowed with multiple sustained-release, pH-responsive, photoresponsive, antibacterial, anti-inflammatory, and pro-angiogenic functions. Representative studies have incorporated silver nanoparticles, usnic acid, copper ions, growth factors, or other bioactive components to combine bacterial control with wound repair (Ficai et al., 2018; Aliakbar Ahovan et al., 2022; Jia et al., 2023; Thang et al., 2023). Electrospun membranes provide a complementary platform because their high porosity, large surface area, and extracellular-matrix-like architecture are suitable for infected-wound dressings. Chitosan/gelatin membranes, berberine-loaded cellulose acetate/gelatin dressings, and quaternized chitosan-modified fibers have all shown antibacterial or wound-healing potential against *P. aeruginosa* infection models (Ficai et al., 2018; Kravanja et al., 2019; Samadian et al., 2020). In the context of the present keyword clusters, these findings explain why hydrogel, electrospinning, chitosan, biocompatibility, and wound healing form a coherent intervention-oriented theme.

Nanotechnology-related studies provide another major explanation for the prominence of antibiofilm agents, antibacterial activity, green synthesis, and material-assisted intervention terms. Silver nanoparticles(AgNPs) exert antimicrobial effects through membrane interaction, membrane disruption, and reactive oxygen species (ROS) generation (Al-Momani et al., 2023); they exhibit a MIC of 1-200 μg/mL against *P. aeruginosa* (de Lacerda Coriolano et al., 2021). Functionalization can substantially modulate their antibiofilm performance, and AgNPs may show synergistic activity with tobramycin or aztreonam by enhancing penetration through the biofilm matrix and damaging bacterial membranes (Habash et al., 2014, 2017; Huma et al., 2020; Solomon et al., 2025). Zinc oxide nanoparticles (ZnONPs) exert antimicrobial activity via electrostatic forces, zinc ion release, and ROS generation (Li et al., 2020; Mendes et al., 2022); at sub-inhibitory concentrations, they significantly inhibit biofilm formation and downregulate QS gene expression (de Celis et al., 2022; Valadbeigi et al., 2023; Konkuri et al., 2024). Furthermore, ZnO-Ag nanocomposites integrate the advantages of both nanoparticles, significantly disrupting pre-formed biofilms through membrane damage and oxidative stress (Alhosani et al., 2025). The recent burst of “green synthesis” further suggests that plant-mediated nanoparticle synthesis has become a current frontier, linking natural-source inhibitors with environmentally friendly antimicrobial material development (Das and Karankar, 2019; Kazmi et al., 2024).

Drug-delivery systems further demonstrate how conventional antibiotics are being repositioned within engineered anti-biofilm platforms. To address the protective role of extracellular DNA (eDNA), DNase I-functionalized nanoparticles have been developed; these systems can eradicate over 99.8% of established biofilms by degrading the matrix scaffold and providing controlled release of ciprofloxacin (Baelo et al., 2015). Polymeric nanoparticles can also improve delivery efficiency through chemical shielding, charge reversal, and magnetic or ultrasound-mediated transport (Wang et al., 2023). For instance, PEGylated tobramycin facilitates antibiotic penetration into the biofilm, exhibiting an efficacy seven times greater than that of free tobramycin (Ding et al., 2024).

Biologically targeted interventions, especially antimicrobial peptides (AMPs) and QS-related inhibitors, provide an additional mechanism-guided direction. AMPs can disrupt membrane potential, destabilize extracellular matrix components, and downregulate QS genes such as lasR and rhlR (Yasir et al., 2018; de Albernaz et al., 2025). Host defense peptides and engineered variants use high cationicity and amphipathicity to penetrate complex biofilm architectures and eliminate embedded pathogens (Pulido et al., 2016; Chen et al., 2018). Nanoparticle-based AMP delivery systems—incorporating silver, polymers, or porous silicon—enhance membrane permeability and suppress efflux pump expression (Kwon et al., 2017; Pal et al., 2019; Chen et al., 2025). Combinations of AMPs with conventional antibiotics can also reduce MBECs, and maintain high efficacy even within the demanding physiological conditions including the cystic fibrosis airway (Dosler and Karaaslan, 2014; Lashua et al., 2016). Taken together, these intervention-oriented clusters suggest that future anti-biofilm therapy will likely depend on multifunctional platforms rather than single-agent antibacterial activity alone.

#### 4.2.3 Clinical translation and efficacy evaluation

The third research dimension identified by keyword clustering was clinical translation and efficacy evaluation. This bibliometric signal was further supported by the supplementary review of PubMed-indexed clinical trials and ClinicalTrials.gov records. Together, these data indicate that the field has begun to move beyond mechanistic and materials-based experimentation, but the clinical evidence base remains substantially less mature than the preclinical literature.

Among published studies, only six interventional trials were directly relevant after screening, and these were concentrated in three settings: chronic wounds, catheter-related bloodstream infection, and cystic fibrosis airway infection. In chronic wound settings, adjunctive strategies such as silver-ion hydrofiber dressing, collagenase ointment (Saranholi et al., 2025), and platelet-rich plasma (Pires et al., 2021) improved clinical or microbiological parameters, although no clear superiority between interventions was demonstrated. In catheter-related bloodstream infection, gentamicin–EDTA lock therapy achieved favorable clinical and microbiological cure rates in catheter-related bloodstream infection (Lebeaux et al., 2025). In cystic fibrosis-associated chronic airway infection, liposomal amikacin for inhalation suspension was noninferior to tobramycin inhalation solution for improving lung function (Bilton et al., 2020), whereas antibiotic therapy guided by biofilm susceptibility testing did not outperform conventional susceptibility-guided treatment (Yau et al., 2015). A pilot crossover study further suggested that higher-frequency intrapulmonary percussive ventilation may disrupt biofilm-associated bacterial behavior and improve pulmonary function (Dingemans et al., 2018). Thus, the published literature indicates that the field has entered clinical experimentation, but not yet clinical consolidation.

The ClinicalTrials.gov dataset broadens this translational picture. Although mature efficacy results remain unavailable for many interventions, the registry-based trial pipeline shows that translational research has clearly expanded beyond conventional antibiotic comparison. The registered studies are concentrated mainly in chronic pulmonary infection, especially cystic fibrosis and non-cystic fibrosis bronchiectasis, with additional activity in surgical-site infection, pressure ulcers, diabetic foot ulcers, burn wounds, and prevention of nosocomial pneumonia. Notably, the intervention portfolio is dominated by nontraditional anti-Pseudomonas strategies, especially bacteriophage-based therapies, alongside biofilm- or virulence-targeting monoclonal antibodies, inhaled nitric oxide formulations, and gallium-based approaches. This pattern strongly supports the keyword finding that the field is shifting toward adjunctive and mechanistically targeted interventions designed to reduce persistence, disrupt biofilm-associated infection, or prevent recurrent pulmonary exacerbations.

Nevertheless, clinical momentum should not be equated with clinical proof. Although several studies are listed as completed or study complete, many have not yet generated mature peer-reviewed efficacy data; one phage trial was discontinued by the sponsor, and several records remain stale or of unclear status. Accordingly, the current translational pipeline is best understood as broadening rather than maturing. In other words, the field now has a larger clinical-development footprint than is apparent from the published literature alone, but this footprint is still weighted toward early-phase, adjunctive, and exploratory intervention strategies rather than validated standard-of-care alternatives.

From a practical perspective, these findings may be useful for both clinicians and researchers. For clinicians, the results highlight the importance of considering biofilm-associated tolerance in persistent or recurrent *P. aeruginosa* infections, particularly in chronic airway, wound-associated, and device-related settings, while also emphasizing that most emerging anti-biofilm interventions remain exploratory rather than established standard-of-care alternatives. For researchers, the keyword co-occurrence, clustering, and keyword burst results provide a data-driven roadmap for future work on MDR evolution, polymicrobial biofilm interactions, quorum-sensing regulation, functional antibacterial materials, green-synthesized nanoparticles, antimicrobial peptides, and phage- or virulence-targeted therapies. The gap between abundant preclinical studies and limited mature clinical evidence further underscores the need for standardized biofilm models, clinically relevant endpoints, pharmacokinetic/pharmacodynamic assessment, and multicenter clinical validation.

### 4.3 Limitation

This study has several inherent limitations. First, the bibliometric analysis relied primarily on WoSCC and Scopus, while PubMed and ClinicalTrials.gov were used only for the supplementary clinical-trials review; therefore, relevant studies indexed in other databases may still have been missed. Second, the exclusion of non-English literature may have introduced geographic and linguistic bias. Additionally, citation-based indicators are affected by citation latency, meaning that publications from 2024–2025 may not yet reflect their full academic impact. Finally, bibliometric mapping can identify thematic structures and collaborative networks, but it does not directly evaluate the biological or clinical efficacy of the antimicrobial strategies discussed.

## 5 Conclusion

This study provides a bibliometric overview of global research on *Pseudomonas aeruginosa* biofilm-mediated drug resistance from 2014 to 2025, complemented by a supplementary review of published clinical studies and a registry-based overview of interventional trials. The results show that this field has grown steadily and evolved toward three major directions: resistance evolution and pathogenic synergy, novel anti-biofilm interventions, and clinical translation. Although translational research is expanding, especially in chronic airway and wound-associated infections, robust clinical evidence remains limited. Methodologically, the findings are subject to limitations related to database coverage, citation latency, possible language or indexing bias, and the descriptive nature of bibliometric mapping. However, overall, this study outlines the knowledge structure, research hotspots, and emerging trends of the field, and offers a useful reference for future studies on anti-biofilm and anti-resistance strategies.

## Data Availability

The original contributions presented in the study are included in the article/**Supplementary Material**. Further inquiries can be directed to the corresponding author.
