# Supplementary material for: Mapping the knowledge landscape of Pseudomonas aeruginosa biofilm-mediated drug resistance: a bibliometric analysis and clinical trial landscape overview
Source: Front Cell Infect Microbiol. 2026 Jun 1;16:1830404. doi: 10.3389/fcimb.2026.1830404 (PMC13265391; doi:10.3389/fcimb.2026.1830404)
Supplement: Supplementary file 1 [file Table1.docx]

**Table S1.** **Search strategies used for the bibliometric analysis and supplementary clinical-trial overview**

| **Database** | **Search strings & conditions** |
| --- | --- |
| WoSCC | TS = ("*Pseudomonas aeruginosa*" OR "*P. aeruginosa*") AND ("Antimicrobial resistance*" OR "Drug Resistance*" OR "Antibiotic Resistance" OR "Antimicrobial Drug Resistance*" OR "Drug Antimicrobial Resistance*" OR "antibiotic resistance genes" OR "drug-resistance") AND ("Biofilm*" OR "antibiofilm" OR "bacterial biofilm*") |
| Scopus | TITLE-ABS-KEY(("*Pseudomonas aeruginosa*" OR "*P. aeruginosa*") AND ("Antimicrobial resistance*" OR "Drug Resistance*" OR "Antibiotic Resistance" OR "Antimicrobial Drug Resistance*" OR "Drug Antimicrobial Resistance*" OR "antibiotic resistance genes" OR "drug-resistance") AND ("Biofilm*" OR "antibiofilm" OR "bacterial biofilm*")) |
| PubMed | ("*Pseudomonas aeruginosa*"[Title/Abstract] OR "*P. aeruginosa*"[Title/Abstract]) AND (biofilm*[Title/Abstract] OR antibiofilm[Title/Abstract] OR "bacterial biofilm*"[Title/Abstract])  **Article type**: Clinical Trial |
| ClinicalTrials.gov | **Condition or disease**: *Pseudomonas aeruginosa*  **Study type**: Interventional Studies |

**Timespan: 2014-2025**
