# Supplementary material for: Mapping the knowledge landscape of Pseudomonas aeruginosa biofilm-mediated drug resistance: a bibliometric analysis and clinical trial landscape overview
Source: Front Cell Infect Microbiol. 2026 Jun 1;16:1830404. doi: 10.3389/fcimb.2026.1830404 (PMC13265391; doi:10.3389/fcimb.2026.1830404)
Supplement: Supplementary file 2 [file Table2.docx]

**Table S2. PICOS-informed eligibility criteria for the supplementary review of published clinical studies**

| **Element** | **Eligibility criteria** |
| --- | --- |
| **Population** | Human participants in clinical settings involving confirmed or clinically suspected Pseudomonas aeruginosa infection, colonization, or biofilm-associated persistence. |
| **Intervention** | Clinical interventions intended to treat, reduce, disrupt, prevent, or evaluate P. aeruginosa-related infection, colonization, biofilm-associated persistence, or antimicrobial treatment response. |
| **Comparator** | Standard care, placebo, active comparator, conventional treatment, baseline comparison, or no direct comparator for early-phase or single-arm studies. |
| **Outcomes** | At least one clinically or microbiologically relevant outcome directly related to P. aeruginosa, biofilm behavior, antimicrobial response, or infection control. |

| **Study design** | Published human clinical trials or prospective interventional clinical studies indexed in PubMed, including randomized controlled trials, double-blind or single-blind trials, crossover trials, non-inferiority trials, phase I/II trials, pilot interventional studies, and single-arm prospective interventional studies. |
| --- | --- |
