# Supplementary material for: Mapping the knowledge landscape of Pseudomonas aeruginosa biofilm-mediated drug resistance: a bibliometric analysis and clinical trial landscape overview
Source: Front Cell Infect Microbiol. 2026 Jun 1;16:1830404. doi: 10.3389/fcimb.2026.1830404 (PMC13265391; doi:10.3389/fcimb.2026.1830404)
Supplement: Supplementary file 3 [file Table3.docx]

**Table S3. Characteristics and methodological quality of published clinical studies related to *Pseudomonas aeruginosa* biofilm-associated infection or persistence**

| **Study** | **Study Design** | **Population / Setting** | **Intervention** | **Comparator** | **Main Outcomes** | **Key Findings** | **Study Quality Assessment Tools** | **Risk of Bias** |
| --- | --- | --- | --- | --- | --- | --- | --- | --- |

| (Saranholi et al., 2025) | Randomized controlled trial | Patients with superficial infection in venous ulcers (34 patients; 56 ulcers) | Silver-ion hydrofiber dressing | Collagenase ointment | Clinical signs of infection, bacterial load, proportion of biofilm-forming bacteria, clonal identity | Both treatments improved infection signs and reduced bacterial load; the proportion of biofilm-forming bacteria decreased over time but showed no significant difference between groups. | RoB 2：individually randomized, parallel-group trial version | Low |
| --- | --- | --- | --- | --- | --- | --- | --- | --- |
| (Lebeaux et al., 2025) | Prospective Phase I/II clinical trial | Adults with long-term central venous catheter-related bloodstream infections (n=8) | Gentamicin + EDTA-Na₂ lock therapy | No direct comparator (single-arm interventional study) | Clinical and microbiological cure at day 40; safety outcomes | Six of seven evaluable patients achieved clinical and microbiological cure; one recurrence occurred in a patient with *P. aeruginosa*. | NIH Quality Assessment Tool for Before-After Studies With No Control Group | Fair |
| (Pires et al., 2021) | Randomized controlled trial | Patients with chronic venous ulcers (n=36) | Platelet-rich plasma (PRP) + petroleum jelly gauze | Petroleum jelly gauze alone | Infection improvement, bacterial load, antibiotic susceptibility, biofilm formation ability | *P. aeruginosa* was detected in 39% of samples and all isolates were biofilm-forming; PRP treatment did not increase bacterial burden. | RoB 2：individually randomized, parallel-group trial version | Some concerns |
| (Bilton et al., 2020) | Multicenter randomized non-inferiority trial | Patients with cystic fibrosis chronically infected with *P. aeruginosa* | Liposomal amikacin for inhalation (ALIS) once daily | Tobramycin inhalation solution (TIS) twice daily | Change in FEV1, respiratory symptoms (CFQ-R), adverse events | ALIS demonstrated non-inferiority to TIS in improving lung function and had a comparable safety profile. | RoB 2：individually randomized, parallel-group trial version | Some concerns |
| (Dingemans et al., 2018) | Pilot crossover clinical study | Patients with cystic fibrosis chronically colonized with *P. aeruginosa* (n=4) | Intrapulmonary percussive ventilation (IPV) at two frequencies | Autogenic drainage | Pulmonary function (FEV1, FVC), bacterial gene expression | Higher-frequency IPV improved lung function and increased expression of planktonic bacterial markers, suggesting disruption of biofilm behavior. | RoB 2：crossover trials version | Some concerns |
| (Yau et al., 2015) | Multicenter randomized double-blind clinical trial | Cystic fibrosis patients with pulmonary exacerbations due to *P. aeruginosa* (39 patients; 74 exacerbations) | Antibiotic therapy guided by biofilm susceptibility testing | Antibiotic therapy guided by conventional susceptibility testing | ≥3-log reduction in sputum bacterial density; clinical outcomes | No significant difference between groups in bacterial density reduction or clinical outcomes. | RoB 2：individually randomized, parallel-group trial version | Low |
