# Supplementary material for: Mapping the knowledge landscape of Pseudomonas aeruginosa biofilm-mediated drug resistance: a bibliometric analysis and clinical trial landscape overview
Source: Front Cell Infect Microbiol. 2026 Jun 1;16:1830404. doi: 10.3389/fcimb.2026.1830404 (PMC13265391; doi:10.3389/fcimb.2026.1830404)
Supplement: Supplementary file 4 [file Table4.docx]

**Table S4. Registered interventional trials from ClinicalTrials.gov included in the trial landscape overview**

| **Registry ID** | **Study Design** | **Population / Setting** | **Intervention** | **Comparator** | **Primary or Major Outcomes** | **Relevance to *P. aeruginosa*** | **Current Status** |
| --- | --- | --- | --- | --- | --- | --- | --- |

| (ClinicalTrials.gov: NCT07088926) | Interventional placebo-controlled efficacy/safety trial | Participants aged ≥12 years with bronchiectasis and chronic *P. aeruginosa* colonization | AZD0292 (IV bispecific anti-Psl/PcrV monoclonal antibody) | Placebo | Annualized rate of moderate-to-severe pulmonary exacerbations; safety; PK | Registry-listed ongoing trial evaluating whether AZD0292 reduces pulmonary exacerbations in bronchiectasis patients chronically colonized with *P. aeruginosa*. | Recruiting |
| --- | --- | --- | --- | --- | --- | --- | --- |
| (ClinicalTrials.gov: NCT06998043) | Phase 2b randomized placebo-controlled trial | Cystic fibrosis subjects with chronic *P. aeruginosa* lung infection | BX004 nebulized bacteriophage therapy | Placebo | Change in sputum *P. aeruginosa* burden at 8 weeks | Registry-listed phage trial designed to test whether 8 weeks of BX004 reduces sputum bacterial burden on top of background CF therapy. | Discontinued by sponsor |
| (ClinicalTrials.gov: NCT06663176) | Phase 1/2a multicenter randomized open-label trial | Adults with non-cystic fibrosis bronchiectasis and confirmed *P. aeruginosa* respiratory infection | Nebulized RESP302 / RESP303 nitric oxide formulations | Active formulation comparison | Safety, tolerability, PK, exploratory efficacy | Registry-listed nitric oxide study evaluating safety and exploratory efficacy of inhaled NO formulations in non-CF bronchiectasis with *P. aeruginosa*. | Recruiting |
| (ClinicalTrials.gov: NCT06319235) | Interventional safety/efficacy trial | Patients with surgical site infection caused by *P. aeruginosa* and/or *S. aureus* | DUOFAG® phage cocktail (IMP) | Placebo | Safety; time to commencement of healing; clinical and microbiological change | Registry-listed recruiting phage cocktail trial in surgical wound infection, relevant to mixed-species wound biofilm settings involving *P. aeruginosa*. | Recruiting |
| (ClinicalTrials.gov: NCT06159725) | Safety/tolerability trial | People with cystic fibrosis and persistent infection | CMTX-101 (biofilm-disrupting monoclonal antibody) | Placebo | Adverse events, serious adverse events, PK, immunogenicity | Completed registry-listed first-in-patient study of a bacterial biofilm-disrupting monoclonal antibody intended as adjunctive therapy to standard antibiotics. | Completed |
| (ClinicalTrials.gov: NCT05616221) | Phase 2 multicenter double-blind randomized placebo-controlled trial | Subjects with non-cystic fibrosis bronchiectasis and chronic pulmonary *P. aeruginosa* infection | Inhaled AP-PA02 bacteriophage cocktail | Placebo | Change in sputum *P. aeruginosa* density; safety; phage kinetics | Completed phage trial evaluating inhaled AP-PA02 in chronic pulmonary *P. aeruginosa* infection outside the CF setting. | Completed |
| (ClinicalTrials.gov: NCT05453578) | Phase 1b/2 dose-escalation and placebo-controlled trial | Adults with cystic fibrosis chronically colonized with *P. aeruginosa* | WRAIR-PAM-CF1 intravenous bacteriophage mixture | Placebo | Change in quantitative sputum *P. aeruginosa* counts; DOOR rank; safety | Completed trial evaluating the microbiological activity and safety of IV phage therapy in clinically stable CF subjects with chronic *P. aeruginosa* colonization. | Completed |
| (ClinicalTrials.gov: NCT05010577) | Phase 1b/2a placebo-controlled trial | Cystic fibrosis patients with chronic *P. aeruginosa* pulmonary infection | BX004-A nebulized bacteriophage therapy | Placebo | Safety and tolerability; exploratory reduction in sputum bacterial load | Completed inhaled phage trial focused primarily on safety, with exploratory assessment of bacterial-load reduction. | Completed |
| (ClinicalTrials.gov: NCT04815798) | Adjunctive placebo-controlled interventional trial | Patients with pressure ulcers colonized with *P. aeruginosa*, *S. aureus*, or *K. pneumoniae* | BACTELIDE topical bacteriophage-loaded microcapsule spray + standard of care | Placebo + standard of care | Treatment-emergent adverse events; tolerability | Registry-listed topical phage trial in chronic wound care, relevant to polymicrobial wound biofilm contexts that include *P. aeruginosa*. | Unknown status / registry stale |
| (ClinicalTrials.gov: NCT04803708) | Phase I/IIa interventional trial | Patients with diabetic foot ulcers with *P. aeruginosa*, *S. aureus*, or *Acinetobacter* infection | TP-102 topical bacteriophage therapy | No direct comparator reported in the registry export | Local/systemic adverse events; serious adverse events | Completed early-phase topical phage study in diabetic foot ulcers, mainly focused on safety and tolerability. | Completed |
| (ClinicalTrials.gov: NCT04684641) | Phase 2 placebo-controlled trial | Cystic fibrosis subjects with chronic *P. aeruginosa* infection | YPT-01 phage therapy | Placebo | Change in sputum *P. aeruginosa* culture titers at day 14; safety | Completed CF phage trial designed to determine whether phage therapy reduces sputum bacterial load. | Completed |
| (ClinicalTrials.gov: NCT04596319) | Phase 1/2 double-blind randomized placebo-controlled SAD/MAD trial | Subjects with cystic fibrosis and chronic pulmonary *P. aeruginosa* infection | Inhaled AP-PA02 | Placebo | Safety, tolerability, phage recovery profile | Completed early-phase inhaled phage study in CF, assessing tolerability and pharmacodynamic recovery of phages after dosing. | Completed |
| (ClinicalTrials.gov: NCT04323475) | Adjunctive interventional trial | Burn patients with wounds susceptible to or infected by *P. aeruginosa*, *S. aureus*, or *K. pneumoniae* | Phage Cocktail-SPK spray + standard therapy | Standard therapy / active wound care comparator | Safety and tolerability | Registry-listed phage adjunct trial in burn wound infection prevention/treatment, relevant to wound biofilm management. | Unknown status / registry stale |
| (ClinicalTrials.gov: NCT03669614) | Phase 1/2a randomized double-blind dose-ascending study | Healthy adults and cystic fibrosis subjects with *P. aeruginosa* infection | Inhaled AR-501 (gallium citrate) | Inhaled placebo | Safety; PK; exploratory efficacy | Registry-listed inhaled gallium study evaluating a non-antibiotic anti-*Pseudomonas* strategy with persistence/biofilm relevance. | Results overdue / registry status unclear |
| (ClinicalTrials.gov: NCT02696902) | Placebo-controlled efficacy/safety trial | Mechanically ventilated subjects at risk for nosocomial *P. aeruginosa* pneumonia | MEDI3902 (anti-Psl/PcrV bispecific monoclonal antibody) | Placebo | Incidence of nosocomial pneumonia caused by *P. aeruginosa*; safety | Completed biologic trial assessing prevention of *P. aeruginosa* pneumonia through a Psl/PcrV-targeted strategy. | Completed |
| (ClinicalTrials.gov: NCT02354859) | Phase 2 interventional trial | Patients with cystic fibrosis | IV gallium nitrate | Normal saline | Proportion of participants with ≥5% relative improvement in FEV1 from baseline to day 28 | Completed gallium trial evaluating a non-traditional antipseudomonal strategy in CF. | Completed |
| (ClinicalTrials.gov: NCT02255760) | Phase 1 randomized double-blind placebo-controlled dose-escalation study | Healthy adults | MEDI3902 | Placebo | Treatment-emergent adverse events; serious adverse events; PK | Completed first-in-human study establishing safety and PK of MEDI3902 before patient-focused efficacy testing. | Study complete |
| (ClinicalTrials.gov: NCT02116010) | Interventional phage therapy trial | Burn patients with *P. aeruginosa* or *E. coli* wound infection | Topical phage cocktail directed against *P. aeruginosa* or *E. coli* | Standard of care (silver sulfadiazine) | Time to persistent bacterial reduction/eradication | Registry-listed phage therapy study in burned patients, representing an early clinical translation attempt for phage-based wound infection control. | Unknown status |
